# Cytotoxic Effect of *Andrographis paniculata* Associated with 2-Aminoethyl Dihydrogen Phosphate in Triple-Negative Breast Cells

**DOI:** 10.3390/cimb46010034

**Published:** 2024-01-05

**Authors:** Rosa Andrea Nogueira Laiso, Julia Carolina Ferreira, Rose Eli Grassi Rici, Laertty Garcia de Sousa Cabral, Durvanei Augusto Maria

**Affiliations:** 1 Laboratory of Development and Innovation, Butantan Institute, Sao Paulo 05585-000, Brazil; laertty.c@usp.br; 2 Postgraduate Program in Structural and Functional Interactions in Rehabilitation, University of Marilia, UNIMAR, Marilia 17525-902, Brazil; jucarolina18@gmail.com (J.C.F.); roseeli@usp.br (R.E.G.R.); 3 Postgraduate Program in Domestic and Wild Animals, Faculty of Veterinary Medicine and Zootechnics, FMVZUSP, University of São Paulo, Sao Paulo 14049-900, Brazil; 4 Faculty of Medicine, FMUSP, University of Sao Paulo, Sao Paulo 14049-900, Brazil

**Keywords:** Andrographis paniculate, natural bioactives, monophosphoester, cancer, treatment

## Abstract

Cancer stands out as a major global public health concern and a significant impediment to increasing life expectancy worldwide. Natural bioactives derived from plants are renowned for their efficacy in treating various types of cancer. *Andrographis paniculata* (Burm.f.) is a well-known plant traditionally employed in diverse medical systems across the globe. The 2-AEH_2_P monophosphoester, a molecule intricately involved in phospholipid turnover, demonstrates antiproliferative effects across a broad spectrum of cancer types. This study aims to assess the antitumor, antiproliferative, and pharmacological effects of andrographolide at different concentrations, both individually and in conjunction with 2-aminoethyl dihydrogen phosphate. The cytotoxicity of the treatments was evaluated using the colorimetric MTT method, cell cycle phases, mitochondrial electrical potential, and markers expression via flow cytometry, while the pharmacological effects were assessed using SynergyFinder software 3.0. Treatments with *A. paniculata*, isolated at concentrations of 10%, 30%, and 50% of andrographolide, induced cell death in tumor cells, resulting in a reduction in mitochondrial electrical potential and alterations in cell cycle phases, particularly a decrease in the population of MDA MB-231 cells in the G0/G1 phase. The combination treatments exhibited significant cytotoxicity toward tumor cells, with minimal toxicity observed in normal fibroblast cells FN1. This led to a reduction in mitochondrial electrical potential and cell cycle arrest in the S phase for MDA MB-231 cells. Across all concentrations, the combined treatments demonstrated a synergistic pharmacological effect, underscoring the efficacy of the association. There was a change in the markers involved in cell death, such as p53, caspase 3, Bcl-2, and cytochrome c, suggesting the induction of regulated cell death. Markers associated with progression and proliferation, such as cyclin D1 and p21, corroborate the findings for cytotoxicity and cell cycle arrest.

## 1. Introduction

Cancer can be defined as a collection of functional capabilities acquired by human cells, resulting in neoplastic growth due to the accumulation of mutations that drive disordered proliferation and invasive capacity in tissues and organs. These capabilities are pivotal for the initiation, maintenance, and progression of tumors [1,2]. Considering all these characteristics, cancer is currently recognized as one of the major global public health challenges and a significant impediment to the improvement of life expectancy worldwide. It holds the fourth position in premature deaths [3,4,5].

In the world, breast cancer is the leading global cause of incidence, accounting for 11.7% of total cases. Breast cancer incidence rates are rapidly increasing in low- and middle-income countries, such as those in South America, Africa, and Asia. This rise in cases is associated with population aging, changes in behavior and lifestyle, and overdiagnosis linked to the widespread use of mammographic screening [3,4].

Natural bioactives of plant origin are well known and widely utilized due to their effectiveness in treating various types of cancer. They exert diverse effects in the body, inhibiting tumor growth and mitigating the side effects of chemotherapy and radiotherapy [6,7]. Plants encompass a multitude of compounds with biological activities, including bioactive phytochemicals, flavonoids, polyphenols, saponins, triterpenoids, alkaloids, glycosides, and phenols, for example. Some of these bioactivities can attenuate the proliferation of tumor cells through various mechanisms, acting on cell cycle checkpoints and promoting apoptosis by activating initiator and executor caspases [8,9,10].

*Andrographis paniculata* (Burm.f.) is renowned for its intensely bitter taste [11]. This herb has been traditionally used in various systems of medicine worldwide. The plant is reported to contain diterpenoids, flavonoids, and steroids [12]. In the last decade, numerous studies have unveiled a range of properties and activities, including antithrombotic, anti-inflammatory, antiviral, immunostimulant, hypotensive, antihyperglycemic, and anticancer effects [13,14].

Andrographolide, a labdane diterpenoid and the primary constituent of *A. paniculata*, has been widely utilized in Asia as a herbal remedy. Andrographolide demonstrates beneficial effects in inflammation-related conditions, including viral infections, bacterial dysentery, malaria, herpes, fever, laryngitis, rheumatoid arthritis (RA), and cancer [15].

The monophosphoester 2-AEH_2_P utilized by our research group a few years ago has already been characterized, revealing its antitumor potential both in vitro and in vivo. This molecule plays a crucial role in phospholipid renewal, serving as a precursor in the synthesis of membrane phospholipids, such as phosphatidylcholine and phosphatidylethanolamine. Both play essential roles in lipid signaling pathways by acting as ligands or generating intermediate substrates [16,17]. Our research group has consistently reported antiproliferative effects in a diverse array of tumor cell lines. During in vivo studies, the treatment of mice with 2-AEH_2_P has demonstrated antiproliferative effects across a broad spectrum of cancer types, irrespective of the expression profile of resistance genes [18,19].

This current study aims to assess the antitumor, antiproliferative, and pharmacological effects of Andrographolide present in *A. paniculata* at concentrations of 10%, 30%, and 50%, both individually and in combination with 2-aminoethyl dihydrogen phosphate in triple-negative human breast adenocarcinoma cells MDA MB-231 and triple-negative human breast carcinoma cells MDA MB-453.

## 2. Materials and Methods

### 2.1. Obtaining the Crude Extract of Andrographis Paniculate

Andrographis extract samples were obtained from FitoPhos (Fort Lauderdale, FL, USA). The crude extract refers to a solution obtained without purification processing or isolation of any component and is only obtained by macerating the entire plant. The andrographolide samples were commercially obtained, as mentioned earlier, in the following concentrations:

− Andrographis extract (standard)/10.0% andrographolide;
− Andrographis extract (standard)/30.0% andrographolide;
− Andrographis extract (standard)/50.0% andrographolide.

The extracts were obtained from the previous maceration, the dried sample was weighed (2.5 g) in a beaker, and solvent (25 mL) was added according to the assay protocol: ethanol in a ratio of 1:10 (g/v). Subsequently, the mixture was placed on a heating plate covered with aluminum foil and stirred for 30 min at a temperature of 40 °C. After the extracts were filtered through filter paper and centrifuged at 3000 rpm for 20 min, the supernatants were concentrated using a rotary evaporator (Buchi R-114), transferred to amber vials, and stored in a freezer (−18 °C) for subsequent lyophilization [20,21].

The lyophilized samples obtained from the company FitoFos, containing Andrographis at concentrations of 10%, 30%, and 50% andrographolide, were solubilized in PBS (phosphate-buffered saline) to obtain samples at concentrations of 14 mg/mL of *A. paniculata* containing the respective concentrations of andrographolide.

### 2.2. Formulation of 2aminoethyl Dihydrogen Phosphate

The 2-AEH_2_P used in this study was prepared as previously published [19]. The 1M stock solution was prepared in ultrapure water and stored at room temperature for in vitro experiments. It has been previously formulated in our laboratory for earlier studies and has proven to be stable.

### 2.3. Cell Culture

The cell lines utilized in this study included the human triple-negative breast adenocarcinoma MDA-MB-231 (ATCC^®^ HTB-26™) and human triple-negative breast carcinoma MDA-MB-453 (ATCC^®^ HTB-131™), both commercially obtained from the American Type Culture Collection. Additionally, the normal human fibroblast cell line FN1 (CAPPesq HCFMUSP No. 921/06) was isolated by Professor Dr. Durvanei Augusto Maria and its corresponding record deposited with CAPPesq at the Hospital das Clínicas of the University of São Paulo, under the mentioned registration number. The cells were cultured in RPMI-1640 medium (LGC Biotecnologia, Cotia, SP, Brazil), supplemented with 10% fetal bovine serum, 100 units/mL of penicillin G, and 100 μg/mL of streptomycin. Cell incubation took place at 37 °C in a CO_2_-injected incubator.

### 2.4. Determination of Cytotoxic Activity using the MTT Colorimetric Method

The tumor and normal cells were incubated in 96-well plates at a density of 1 × 10^5^ cells/mL for 24 and 48 h. Subsequently, the treatments were administered using Andrographis at concentrations of 10%, 30%, and 50% of andrographolide. For combination treatments, the concentration corresponding to half of the IC_50_ of fixed 2-AEH_2_P in 10 mM was utilized, and Andrographis concentrations of 10%, 30%, and 50% were established to evaluate the pharmacological effect of this combination. After the designated treatment period, the supernatant was aspirated, and 100 μL of MTT at a concentration of 5 mg/mL (Calbiochem—Darmstadt, Germany) was added to the plates. The plates were then incubated for 3 h at 37 °C in an atmosphere containing 5% CO_2_. Subsequently, the content was removed, and 200 μL of methanol was added to dissolve the formazan crystals. Absorbance was measured at a wavelength of 540 nm using a microplate reader.

### 2.5. Analysis of Cell Cycle Phases and Fragmented DNA using Flow Cytometry

Tumor and normal cells were treated at an IC_50_ concentration for 24 h. Treated and control cells were trypsinized and centrifuged at 1200 rpm for 5 min. Subsequently, the resulting pellet was resuspended in 1 mL of cold buffer through dripping, followed by the addition of 3 mL of absolute ethanol. The samples were then stored at −20 °C for 24 h. Afterward, the samples were centrifuged at 1500 rpm for 10 min and resuspended in 200 μL of FACS buffer (fluorescence-activated cell sorting), 20 μL of Triton X-100 (Sigma-Aldrich—San Luis, MO, USA), 50 μg/mL of propidium iodide (Sigma-Aldrich), and 1 μL of RNAse (200×), and kept for 30 min at room temperature protected from light. The prepared samples were then transferred to cytometry tubes and analyzed using a FACScanto flow cytometer (BD), capturing 10,000 events. The resulting histograms were acquired and analyzed using ModFit LT 5.0 software.

### 2.6. Mitochondrial Electrical Potential using Flow Cytometry

Tumor and normal cells underwent a 24 h treatment at an IC_50_ concentration. Following treatment, cells were trypsinized and centrifuged at 1200 rpm for 5 min, the supernatant was discarded, and the cells were resuspended in a complete medium containing MitoRed™ (Sigma-Aldrich, USA) at a concentration of 200 nM. Subsequently, the samples were incubated in an oven at 37 °C with 5% CO_2_ for 30 min. After incubation, the tubes were centrifuged, the supernatant was discarded, and the pellet was resuspended in 200 μL of FACS flow buffer. The analysis was conducted using a FACScanto flow cytometer (BD), capturing 10,000 events, and the resulting histograms were acquired and analyzed using FCS Express^TM^ software “https://www.thermofisher.com/order/catalog/product/br/pt/A48515”, accessed on 22 December 2023.

### 2.7. Evaluation of the Number of Cells Expressing Markers Involved in Apoptosis and Progression using Flow Cytometry

Following the indicated treatments, 100 µL aliquots of the MDA-MB 231 tumor cells at a concentration of 10^6^ cells/mL were incubated with Triton X-100 (0.1% final) for 30 min. The mixture was then centrifuged at 1500 rpm for 5 min, and the pellet was resuspended in 200 µL of FACS buffer and incubated with 1 µg of specific antibody to either caspase 3, p53, p21, Bcl-2, and cytochrome c conjugated with phycoerythrin (PE) for 1 h at 4 °C. Antibodies to markers involved in proliferation and progression of cell cycle phases, such as cyclin D1 conjugated to phycoerythrin (PE), were also used. The cells were then centrifuged at 1500× *g* rpm and washed with PBS, and the pellet was resuspended in 200 µL of FACS buffer containing 0.1% paraformaldehyde. The analysis of marker expression was performed in a FACScanto flow cytometer–BD (Franklin Lakes, NJ, USA) at FLH-1 fluorescence intensity (10,000 events), and DotPlots were acquired and analyzed using Cell-Quest-DB 2.0 software.

### 2.8. In Silico Analysis of the Pharmacological Effect of the Associations

To assess the potential synergy of the formulations, a matrix study was conducted with Andrographis at concentrations of 10%, 30%, and 50% in association with 2-AEH_2_P. The combination matrix was tested on MDA MB-231 triple-negative human breast adenocarcinoma cells. SynergyFinder 2.0 software quantified the degree of synergy as the excess over the multiplicative effect of single drugs, assuming they acted independently (Bliss). The following higher-order formulations were employed to quantify the pharmacological effect of drug combinations (S) for the multiple-drug combination effect measured between the two drugs:

$$S_{BLISS}=E_{A,B}-\left(E_{A}+E_{B}\right)\;SynergyScore=\frac{-10_{g\left(p\right)}}{\log\left(0.05\right)}\times \frac{t}{\left|t\right|}$$

### 2.9. Statistical Analyses

All values obtained are expressed as mean ± standard deviation. After obtaining individual values for each treated and control cell line, the results were tabulated and analyzed using GraphPad Version 5.0 and GraphPad Prism Version 7.0. Data analysis involved comparing two or more groups with nonparametric distribution using analysis of variance (ANOVA), followed by the Tukey–Kramer multiple comparison test, considering *p* < 0.05 as the critical level for significance.

## 3. Results

### 3.1. Cytotoxic Potential of A. paniculata and Its Association with 2-AEH_2_P

**Figure 1:**
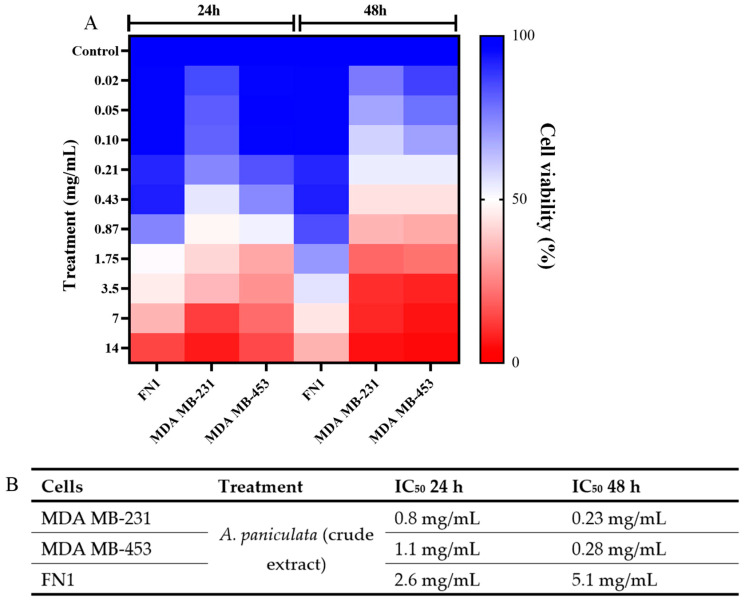
Determination of cytotoxicity in tumor and normal cells using the MTT colorimetric method. The cells were treated with different concentrations of crude extract of *A. paniculata* in the periods of 24 h and 48 h. (**A**) Heatmap expressed as mean ± SD of three independent experiments with *A. paniculata* treatment over 24 and 48 h periods; (**B**) Table with the IC_50_ values obtained for each cell in the treatment times.

The results of the viability evaluation showed that *A. paniculata* at concentrations of 10%, 30%, and 50% and when associated with 2-AEH_2_P induced cytotoxicity for the studied tumor cells. In the 24 h period, it was possible to observe an IC_50_ value of 0.8 mg/mL for the MDA MB-231 tumor cell, a value of 1.1 mg/mL for the MDA MB-453 cell, and a value of 2.6 mg/mL for the normal FN1 cell (Figure 1A,B. In 48 h of treatments, the IC_50_ values fall for the tumor cells, obtaining values of 0.23 mg/mL for the MDA MB-231 cell and a value of 0.28 for the MDA MB-453 cell (Figure 1B). It is noteworthy that the 48 h IC_50_ value for the normal human fibroblast cell increased, with a value of 5.1 mg/mL, thus increasing the therapeutic index of the treatment, leading to greater specificity for the tumor cell (Figure 1B).

**Figure 2:**
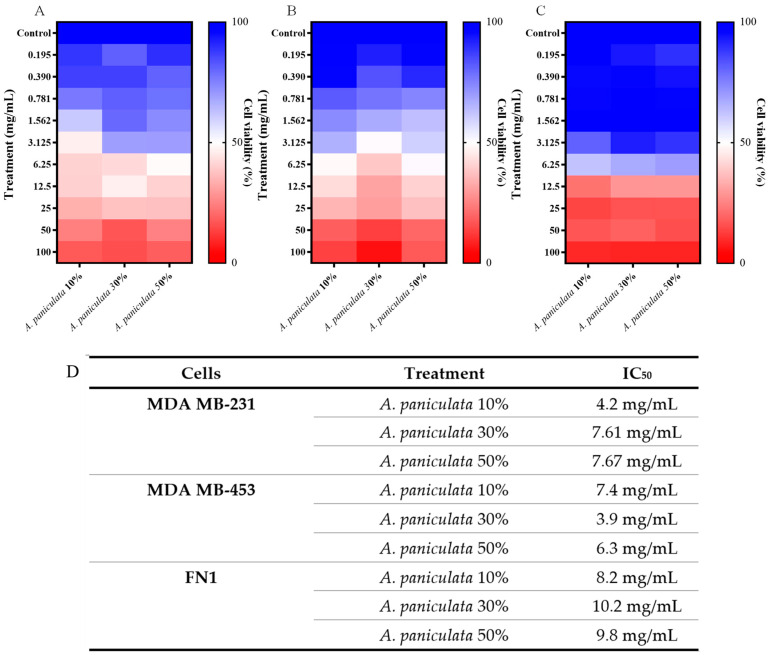
Determination of cytotoxicity in MDA MB-231 and MDA MB-453 tumor cells using the MTT colorimetric method. Cells were treated with different concentrations of Andrographis within 24 h. (**A**) Heatmap expressed as mean ± SD of three independent experiments of *A. paniculata* treatment on MDA MB-231 human triple-negative breast adenocarcinoma tumor cell; (**B**) Heatmap expressed as mean ± SD of three independent experiments of *A. paniculata* treatment on MDA MB-453 human triple-negative breast carcinoma tumor cell; (**C**) Heatmap expressed as mean ± SD of three independent experiments of *A. paniculata* treatment on FN1 fibroblast normal cell; (**D**) Table with IC_50_ values obtained.

The cytotoxicity of *A. paniculata* was determined at concentrations of 10%, 30%, and 50% of andrographolide for both tumor and normal cells. The MDA MB-231 tumor cell showed an IC_50_ value for treatment with *A. paniculata* in a 24 h period of 4.2 mg/mL, 7.61 mg/mL, and 7.67 mg/mL when treated at concentrations of 10%, 30%, and 50%, respectively (Figure 2A–C). The MDA MB-453 tumor cell, under the same conditions described above, presented IC_50_ values of 7.4 mg/mL, 3.9 mg/mL, and 6.3 mg/mL for treatments with *A. paniculata* at concentrations of 10%, 30%, and 50%, respectively (Figure 2B,C).

The FN1 normal human fibroblast cell showed a higher IC_50_ but did not show selectivity in the degrees of concentration and time analyzed. The IC_50_ values obtained for the treatment with *A. paniculata* at concentrations of 10%, 30%, and 50% andrographolide were 8.2 mg/mL, 10.25 mg/mL, and 9.8 mg/mL, respectively (Figure 2C,D).

**Figure 3:**
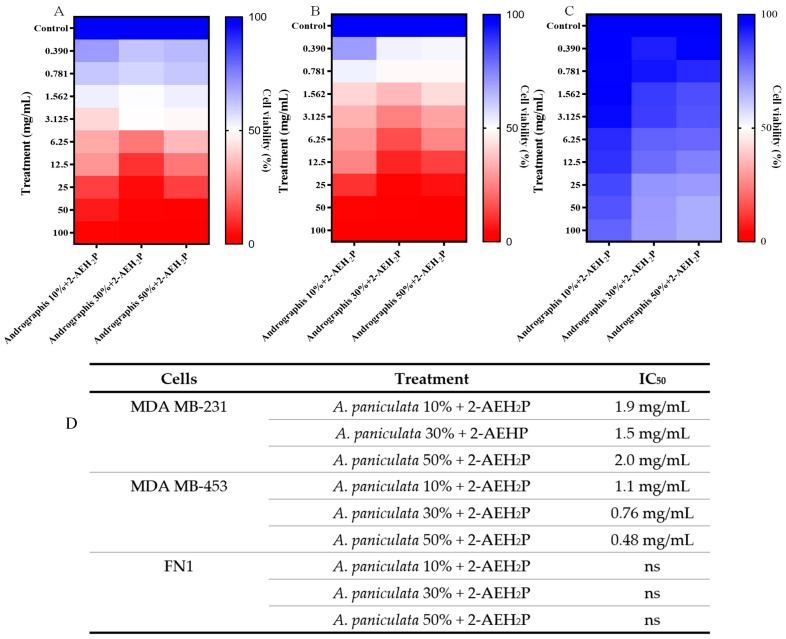
Determination of cytotoxicity in MDA MB-231 tumor cells using the MTT colorimetric method. Cells were treated with different concentrations of *A. paniculata* + 2-AEH_2_P within 24 h. (**A**) Heatmap expressed as mean ± SD of three independent experiments of treatment with *A. paniculata* + 2-AEH_2_P on human triple-negative breast adenocarcinoma tumor cell MDA MB-231; (**B**) Heatmap expressed as mean ± SD of three independent experiments of treatment with *A. paniculata* + 2-AEH_2_P on human triple-negative breast adenocarcinoma tumor cell MDA MB-453; (**C**) Heatmap expressed as mean ± SD of three independent experiments of treatment with *A. paniculata* + 2-AEH_2_P on FN1 fibroblast normal cell; (**D**) Table with IC_50_ values obtained. Ns = not significant.

The cytotoxicity for tumor cells of triple-negative human breast adenocarcinoma MDA MB-231 of the *A. paniculata* formulation associated with 2-aminoethyl dihydrogen phosphate was determined. There was a reduction in the treatment concentrations for *A. paniculata* when associated with 2-AEH_2_P for the MDA-MB-231 tumor cell, with IC_50_ values of 1.9 mg/mL when associated with *A. paniculata* at a concentration of 10% andrographolide, 1.5 mg/mL when associated with *A. paniculata* at 30%, and 2.0 mg/mL when associated with *A. paniculata* at 50% (Figure 3A–C).

A positive effect was observed for the association of *A. paniculata* with 2-AEH_2_P on normal FN1; no marked cytotoxicity was observed. Probably 2-AEH_2_P generated a protective effect for these cells. In the FN1 cell, a 19.6% reduction in viability was observed in the highest concentration tested for *A. paniculata* at 10% associated with 2-AEH_2_P; a reduction of 33.1% and 35% was observed in the highest concentration tested for *A. paniculata* at 30% and 50% associated with 2-AEH_2_P, respectively (Figure 3C,D).

### 3.2. Distribution of Cell Populations Treated with A. paniculata and Its Association with 2-AEH_2_P in Cell Cycle Phases

**Figure 4:**
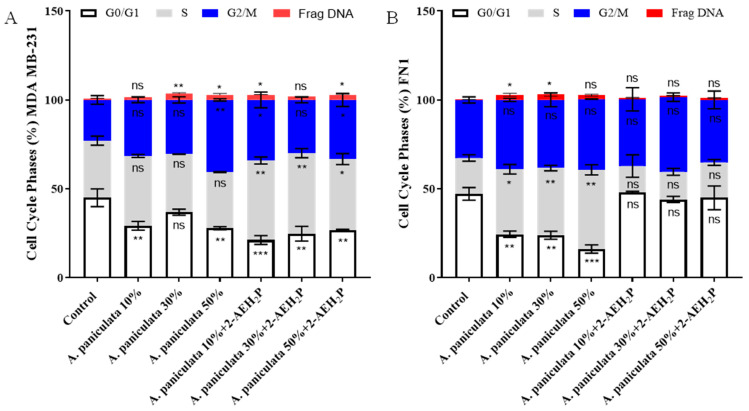
Analysis of cell cycle phases using flow cytometry. The cells were treated with different concentrations of *A. paniculata* alone and associated with 2-AEH_2_P within 24 h. (**A**) Bar graph showing the correlation of the effect on the cell cycle expressed as mean ± SD of three independent experiments of the MDA MB-231 tumor cell; (**B**) Bar graph showing correlation effect on cell cycle expressed as mean ± SD of three independent experiments on normal FN1 cell. Statistical differences were obtained using ANOVA and Tukey–Kramer multiple comparison tests. * *p* < 0.05, ** *p* < 0.01, and *** *p* < 0.001. ns = not significant.

The analysis of the percentage of MDA MB-231 tumor cells in the different phases of the cell cycle when treated with *A. paniculata* at a concentration of 10% and 50% showed a reduction in cells in the G0/G1 phase of the cell cycle by 29.1 ± 3.4% and 36.8 ± 2.3%, respectively (Figure 4A). When associated with 2-AEH_2_P, an arrest in the S phase of the cell cycle was observed in 44.7 ± 2.7%, 45.34 ± 3.6%, and 40.1 ± 4.3% for concentrations of 10%, 30%, and 50% andrographolide of *A. paniculata*, respectively (Figure 4A). There was an increase in fragmented DNA for the isolated treatments at concentrations of 30% (1.45 ± 0.8%) and 50% (3.4 ± 0.8%) andrographolide and in associations of 2-AEH_2_P with *A paniculata* at 10% (2.87 ± 0.2%) and 50% (2.82 ± 0.4%) andrographolide (Figure 4A).

The distribution of FN1 normal human fibroblast cells in cell cycle phases was carried out. There was a stop in the S phase of the cell cycle for treatments with *A paniculata* isolated at concentrations of 10%, 30%, and 50% andrographolide, with values of 36.5 ± 3.7%, 37.7 ± 2.2%, and 44.5 ± 4%, respectively (Figure 4B). No changes were observed in cell destruction in the cell cycle phases for the association of *A. paniculata* with 2-AEH_2_P at any of the tested concentrations (Figure 4B). There was an increase in fragmented DNA for the isolated treatment of *A. paniculata* at concentrations of 10% and 30%, with values of 2.83 ± 1.2% and 3.1 ± 1.4% (Figure 4B).

### 3.3. Evaluation of the Mitochondrial Electrical Potential (ΔΨm) of Treatments with A. paniculata and Its Association with 2-AEH_2_P

**Figure 5:**
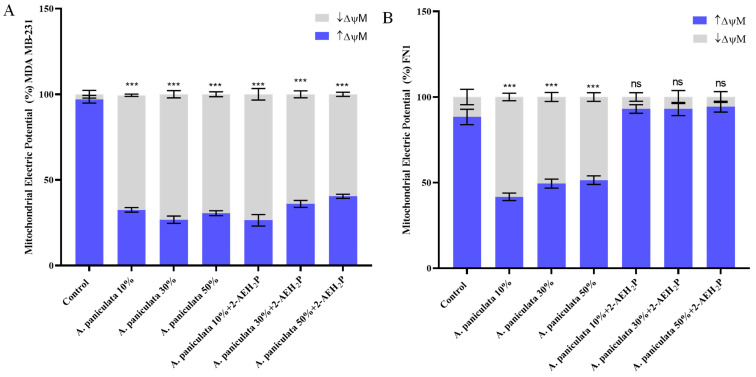
Analysis of mitochondrial electrical potential (ΔΨm) using flow cytometry. Cells were treated with isolated *A. paniculata* and their associations with 2-AEH_2_P for a period of 24 h. (**A**) Bar graph showing ΔΨm expressed as mean ± SD of three independent experiments for the MDA MB-231 tumor cell; (**B**) Bar graph showing the ΔΨm expressed as mean ± SD of three independent experiments for normal cell FN1. Statistical differences were obtained using ANOVA and Tukey–Kramer multiple comparison tests. *** *p* < 0.001. ns = not significant.

After treatment of the triple-negative MDA MB-231 human breast adenocarcinoma cell, it was possible to observe a reduction in the mitochondrial electrical potential for all treatments. There was a reduction of 66.9 ± 0.9% when treated with *A. paniculata* isolated at a concentration of 10% andrographolide, 73.26 ± 31% for treatment at a concentration of 30% andrographolide, and 69.47 ± 2% when treated at a concentration of 50% andrographolide (Figure 5A). A similar result was observed for the treatments of *A. paniculata* when associated with 2-AEH_2_P, with values of 73.6 ± 4.7%, 64 ± 2.8%, and 60.1 ± 1.6% for concentrations of 10%, 30%, and 50% andrographolide, respectively (Figure 5A). 

Similar to the result obtained for the cell cycle, there was only a change in the mitochondrial electrical potential for the normal human fibroblast cell in the isolated treatments of *A. paniculata*, with no reduction in the association with 2-AEH_2_P (Figure 5B). There was a reduction of 58.3 ± 3% for treatment with *A paniculata* at a concentration of 10% andrographolide, 50.6 ± 3.7% when treated at a concentration of 30% andrographolide, and 48.5 ± 3.5% for treatment at 50% andrographolide concentration (Figure 5B).

### 3.4. Analysis of the Mechanisms of Action of Treatment with A. paniculata and Its Association with 2-AEH_2_P

**Figure 6:**
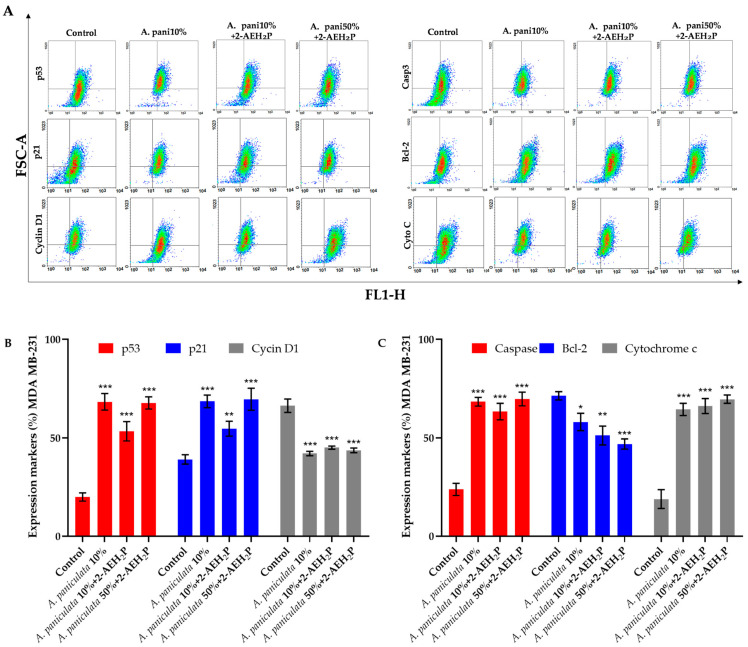
Analysis of marker expression in MDA MB-231 triple-negative human breast cancer tumor cells. Marker expression was quantified using flow cytometry after 24 h of treatment with *A. paniculata* 10% and the combination *A. paniculata* 10%/2-AEH_2_P and *A. paniculata* 50%/2-AEH_2_P. (**A**) Representative density plots showing the distribution of cells with fluorescence intensity; (**B**) Expression of markers p53, p21, and cyclin D1; (**C**) Expression of markers caspase 3, Bcl-2, and cytochrome C. Bar graphs present protein expression levels as mean ± SD from three independent experiments. Values are expressed as mean ± standard deviation (SD) from three independent experiments. Statistical differences were determined using ANOVA multiple comparison tests and Tukey–Kramer. * *p*  <  0.05, ** *p*  <  0.01, and *** *p*  <  0.001.

The treatment of MDA MB-231 tumor cells was carried out with *A. paniculata* extract and combined with 2-AEH_2_P at the IC_50_ concentration obtained for the cell line for 24 h. After the treatment period with *A. paniculata 10%,* a significant increase in the expression of the markers p53 and p21 was observed in MDA MB-231 tumor cells, with a percentage increase of 66.2 ± 4.0% and 67.1 ± 3.1% (Figure 6A,B). Similar results were observed for the combined treatment, where there was an increase in p53 expression when treated with *A. paniculata* 10%/2-AEH_2_P at 52.7 ± 4.1% and 64.5 ± 2.0% for treatment with *A. paniculata* 50%/2-AEH2P. For the p21 protein, an increase in expression was also observed for the combinations, with values of 53.2 ± 2.9% (*A. paniculata* 10%/2-AEH_2_P) and 68.2 ± 4.4% (*A. paniculata* 50%/2-AEH_2_P) (Figure 6B). There was a significant reduction in the expression of cyclin D1 for all treatments. When treated with *A. paniculata* 10%, the value obtained was 41.1 ± 1.0%. For the combination *A. paniculata* 10%/2-AEH_2_P, the value was 45.7 ± 1.2%, and for the combination *A. paniculata* 50%/2-AEH_2_P, it was 41.9 ± 1.1%.

In relation to markers involved in cell death, an increase was observed for all of them after treatments on MDA MB-231 cells. After treatment with *A. paniculata 10%*, the percentage of cells expressing caspase 3 was 64.3 ± 2.6%. For the combination treatment *A. paniculata* 10%/2-AEH_2_P, the obtained value was 58.5 ± 3.8%, and for the combination *A. paniculata* 50%/2-AEH_2_P, it was 64.7 ± 3.3% (Figure 6A–C). The changes in the populations of MDA MB-231 cells after treatment, expressing the antiapoptotic marker Bcl-2, showed a positive response to apoptosis, with a reduction in expression. The reduction values obtained were 55.4 ± 4.1% after treatment with *A. paniculata* 10%. When treated with the combination *A. paniculata* 10%/2-AEH_2_P, the value was 47.0 ± 5.3%, and with *A. paniculata* 50%/2-AEH_2_P, it was 37.1 ± 2.1% (Figure 6C). There was a considerable increase in the release of cytochrome C in MDA MB-231 cells, which was proportional to the reduction in the antiapoptotic protein Bcl-2. After treatment with *A. paniculata* 10%, the obtained value was 57.5 ± 3.5%. When associated, the value was 58.4 ± 3.9% (*A. paniculata* 10%/2-AEH_2_P) and 65.8 ± 2.3% (*A. paniculata* 50%/2-AEH_2_P) (Figure 6C).

### 3.5. Pharmacological Activity of the Association of A. paniculata with 2-AEH_2_P

**Figure 7:**
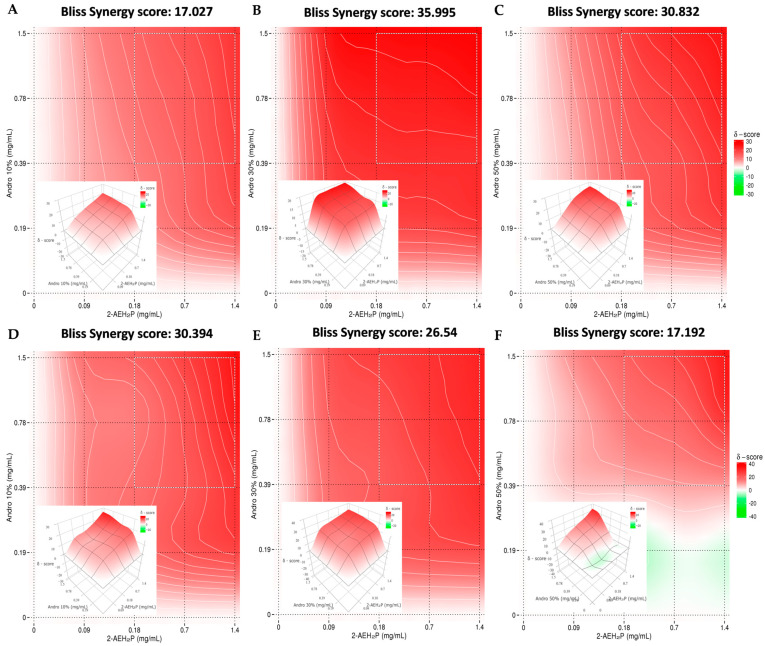

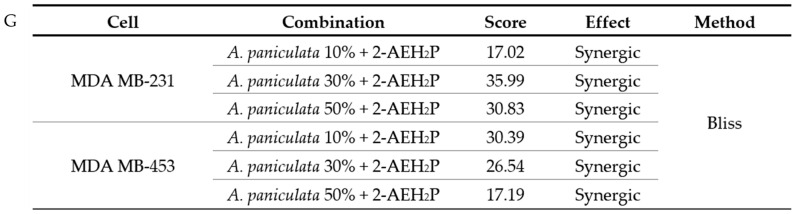
Determination of the pharmacological effect using SynergyFinder software “https://synergyfinder.fimm.fi/”, accessed on 22 December 2023. (**A**) 2D and 3D heat map of the association of *A. paniculata* 10% with 2-AEH_2_P in the MDA MB-231 tumor cell; (**B**) 2D and 3D heat map of the association of *A. paniculata* 30% with 2-AEH_2_P in the MDA MB-231 tumor cell; (**C**) 2D and 3D heat map of the association of *A. paniculata* 50% with 2-AEH_2_P in MDA MB-231 tumor cell; (**D**) 2D and 3D heat map of the association of *A. paniculata* 10% with 2-AEH_2_P MDA MB-453 tumor cell; (**E**) 2D and 3D heat map of the association of *A. paniculata* 30% with 2-AEH_2_P in the MDA MB-453 tumor cell; (**F**) 2D and 3D heat map of the association of A. *paniculata* 10% with 2-AEH_2_P in the MDA MB-453 tumor cell; (**G**) Tables showing the combination of drugs and the synergy values for these combinations. The antagonistic effect is observed in the graph with color pattern between white and green (≤0), and the additive and synergistic effect are observed between white and red (>0 and ≤10 additive; >10 synergistic). Color saturation is proportional to the magnitude of the difference between these values.

A synergistic effect was observed for all associations in both tumor cells, MDA MB-231 and MDA MB-453. In triple negative human breast adenocarcinoma cells MDA MB-231 when treated with the association of 2-AEH_2_P with *A. paniculata* at a concentration of 10% andrographolide of *A. paniculata*, the synergism value obtained was 17.02; for 30% andrographolide, the value was 35.99; when treated with the association at a concentration of 50%, the value was 30.83 (Figure 7A–C).

A similar result was observed for the treatment with the MDA MB-453: for the associated treatment of 2-AEH_2_P with *A. paniculata* at 10%, the synergism value was 30.39; for the treatment at the 30% concentration, the synergism value was 26.54; and for the association at the 50% concentration, it was 17.19 (Figure 7D–F). All treatments proved to be efficient in reducing the viability of tumor cells, and from the calculation coefficient following the Bliss method, these associations have a synergistic effect, where one molecule enhances the action of the other.

## 4. Discussion

Treatments with isolated *A. paniculata* showed cytotoxicity for all the cells tested, whether tumors such as triple-negative human breast adenocarcinoma (MDA-MB-231) or triple-negative human breast carcinoma (MDA-MB-453) and for normal human fibroblast cells (FN1), with arrest in cell cycle phases and a reduction in mitochondrial electrical potential. When associated with the 2-AHE_2_P monophosphoester, a protective effect is observed for normal FN1, not causing accentuated cytotoxicity or modulating the distribution of cells in the phases of the cell cycle and mitochondrial electrical potential, thus improving the therapeutic index by continuing to show antitumor and antiproliferative effects on tumor cells.

*A. paniculata* contains the active phytochemicals that are found primarily in plant parts such as leaves and stems mostly containing diterpenoids, including andrographolide, a labdane diterpenoid and the major constituent; neoandrographolide; 14-deoxy-11; 12-didehydroandrographolide; 14-deoxyandrographolide; isoandrographolide; 14-deoxyandrographolide-19-β-D-glucoside; homoandrographolide; andrographolidegraphan; andrographolidegraphosterin; and stigmasterol [22].

*A. paniculata* extracts, along with their primary diterpenoid component, exhibit compelling effects in restraining cell proliferation, instigating cell cycle arrest, and prompting apoptosis in various cancer cell lines [23,24,25]. Notably, andrographolide, a key component, has demonstrated its efficacy in inducing apoptosis in TD-47 human breast cancer cells by elevating p53, the Bcl-2-associated protein X (Bax), and caspase 3 levels while concurrently suppressing Bcl-2 expression [26]. Furthermore, andrographolide treatment has been shown to elicit cell cycle arrest and apoptotic demise in MDA-MB-231 breast cancer cells [22]. The antitumor potential of andrographolide extends to the modulation of tumor angiogenesis, inhibition of cell proliferation and migration, and disruption of vascular endothelial cell tube formation in vivo.

Antitumor action was identified for several types of cancer, among them, human cervical cancer, inhibiting the viability of Hela cells alone or in combination with the flavonoid taxifolin through the induction of caspase 3-dependent apoptosis and autophagy triggered by ROS [27]. In prostate cancer, it inhibits the growth of prostate cancer cells without affecting the primary prostate epithelial cells. It also induces cell cycle arrest, suppressing the viability and migration of these cells by modulating the expression of important chemokines such as CXCL11, CXC chemokine receptor (CXCR)3, and CXCR7 [28].

In esophageal cancer, it inhibits tumor growth by regulating apoptosis and the NF-κB signaling pathway, increasing radiosensitivity, increasing the expression of cleaved caspase 3 and Bax, and decreasing Bcl-2 and NF-κB levels in vitro [29]. A similar result was observed for the treatment with *A. paniculata* in oral cavity cancer cells, significantly inhibiting oncogenicity and promoting radiosensitivity to oral cancer stem cells ALDH1+CD44+ through the upregulation of microRNA-218 and Bmi1 negative [30].

In recent works, the 2-AEH_2_P monophosphoester showed a cytotoxic character for triple-negative human breast adenocarcinoma cells (MDA-MB-231), with an IC_50_ value 30 times higher for tumor cells; there was a significant decrease in lipoperoxides and an increase in the DNA fragmented and arrested in the G0/G1 phases of cell cycle [19,31]. 2-AEH_2_P also showed therapeutic significance for chronic myeloid leukemia (K562), promoting apoptosis independent of the molecular resistance profile (MDR+) [21,32].

The effectiveness of the association of 2-AEH_2_P has been investigated in association with other compounds, seeking to identify its pharmacological action; with the BR2 tumor penetration peptide, the modulation of proteins involved in cell death, such as p53, Bax, Bad, Bcl-2, caspase 3 and 8, was observed with cytochrome C release and senescence with increased p21 and reduced cyclin D1 and markers of progression and invasiveness, CD44, CD34, CD24 for triple-negative MDA MB-231 human breast adenocarcinoma cells [18].

An important synergistic effect was observed by the association, reducing the concentration of both compounds and increasing the therapeutic index without causing damage to normal cells and possibly minimizing the systemic damage that traditional chemotherapy causes. Although the isolated components of *A. paniculata* show good antitumor and other biological activities, the dose can be reduced when combined with other commonly used drugs or chemotherapeutics [33].

The set of data obtained in the present work demonstrates the antitumor and antiproliferative action of *A. paniculata* in vitro for tumor cells of human triple-negative breast adenocarcinoma and melanoma when isolated, and when associated with monophosphoester 2-AEH_2_P, the effect was reduced cytotoxicity to normal cells, thus showing a protective effect of the compound, increasing the therapeutic index.

## 5. Conclusions

Treatments with *A. paniculata* isolated at concentrations of 10%, 30%, and 50% promoted cell death for tumor cells but showed cytotoxicity for normal FN1 human fibroblast cells. The slightly more efficient concentration compared to control cells was 30% andrographolide. There was a reduction in the mitochondrial electrical potential for all cells tested and an alteration in the cell cycle phases with a reduction in the MDA MB-231 cell population in the G0/G1 phase, and normal FN1 stopped at the G0/G1 phase.

When *A. paniculata* was associated with the 2-AEH_2_P monophosphoester, there was high cytotoxicity for MDA MB-231 tumor cells if it showed toxicity for normal human FN1 cells. The treatments promoted a reduction in the mitochondrial electrical potential for the cells, without altering the potential of the control cells. The only cell that presented alteration in the phases of the cell cycle was MDA MB-231, with an arrest in the S phase of the cell cycle. There was a change in the markers involved in cell death, such as p53, caspase 3, Bcl-2, and cytochrome c, suggesting the induction of regulated cell death. Markers associated with progression and proliferation, such as cyclin D1 and p21, corroborate the findings for cytotoxicity and cell cycle arrest.

The combined treatments at all concentrations showed a synergistic pharmacological effect, evidencing the efficiency of the association.

## Data Availability

Data available in a publicly accessible repository. The data presented in this study are openly available in repository Butantan Institute, reference number RP00490.
